# Research Progress on the Diagnosis and Treatment of Intestinal Fibrosis in Crohn's Disease with Traditional Chinese and Western Medicine

**DOI:** 10.5812/ijpr-163617

**Published:** 2025-10-18

**Authors:** Qili Xiao, Delong Mo, Gang Liu, Yuting Xu, Yan Chen, Chuanjian Lu

**Affiliations:** 1 Department of Proctology, Zhongshan Hospital of Traditional Chinese Medicine Affiliated to Guangzhou University of Traditional Chinese Medicine, Zhongshan 528400, Guangdong Province, China; 2 Department of Gastroenterology, Fangcun Hospital of Guangdong Provincial Hospital of Chinese Medicine, Guangzhou 510370, Guangdong Province, China; 3 State Key Laboratory of Dampness Syndrome of Chinese Medicine, Guangdong Provincial Key Laboratory of Clinical Research on Traditional Chinese Medicine Syndrome, Guangdong Clinical Research Center for Dermatosis in Chinese Medicine, The Second Affiliated Hospital of Guangzhou University of Chinese Medicine/Guangdong Provincial Hospital of Chinese Medicine/ Guangdong Provincial Academy of Chinese Medicine, Guangzhou 510120, China

**Keywords:** Crohn’s Disease, Intestinal Fibrosis, Integrated Chinese and Western Medicine

## Abstract

**Context:**

Intestinal fibrosis, a severe complication of Crohn's disease (CD), arises from multifactorial interactions, including chronic inflammation, genetic predisposition, gut microbiota dysbiosis, and impaired mucosal barrier function.

**Objectives:**

Since intestinal fibrosis often leads to irreversible stenosis and obstruction symptoms, this article will focus on a series of reviews on the progress of traditional Chinese and Western medicine in the diagnosis and treatment of intestinal fibrosis in CD.

**Methods:**

We conducted a systematic literature search in PubMed, CNKI, and Wanfang databases using the keywords: 'Crohn's disease', 'intestinal fibrosis', and 'Traditional Chinese Medicine (TCM)'. The initial search yielded 216 articles. After removing duplicates (n = 146), 70 articles underwent full-text screening based on predefined criteria.

**Results:**

Current diagnostic modalities for CD-related intestinal fibrosis are well-established. However, neither pharmacological agents nor TCM therapies have demonstrated definitive efficacy in reversing fibrosis in clinical settings.

**Conclusions:**

Despite the current absence of clinically approved antifibrotic drugs, this review synthesizes critical advances in mechanistic understanding and emerging therapeutic strategies. We highlight the urgent unmet clinical need and propose integrative medicine as a promising paradigm shift for fibrosis management. While no therapies yet fully reverse established fibrosis, this review provides three key contributions: Systematically maps molecular pathways from inflammation to fibrosis, identifies knowledge gaps in TCM translational research, and proposes a “control-prevent-reverse” framework for future drug development. These insights are timely given the rising CD prevalence in China and increasing NIH funding for antifibrotic research.

## 1. Context

Crohn's disease (CD) is a chronic, relapsing inflammatory bowel disease (IBD) characterized by segmental inflammation of the gastrointestinal tract. According to the global burden of disease (GBD) data (1), China harbors the largest proportion of IBD patients worldwide, and this number is expected to rise further by 2030. The highest incidence rates of CD are reported in North America (20.7 per 100,000) and Europe (12.7 per 100,000) (2). Epidemiological surveys conducted in China between 2010 and 2011 indicate an incidence rate of CD ranging from 0.13 to 1.09 per 100,000 individuals (3). Another study estimates that this incidence rate has nearly doubled over the past three decades (4).

The pathological hallmark of CD is transmural inflammation, with intestinal fibrosis being a key pathophysiological process. This condition can lead to severe complications such as intestinal strictures and obstruction, significantly impairing patients' quality of life and increasing the burden on healthcare systems. It is estimated that approximately 30 - 50% of CD patients will develop intestinal fibrosis within 10 years of diagnosis (5).

Although various studies have explored the mechanisms underlying intestinal fibrosis in CD, its exact pathogenesis and effective prevention and treatment strategies remain elusive. Therefore, this review systematically examines the progress in understanding intestinal fibrosis in CD and explores both conventional and integrative medicine approaches to prevention and treatment (Table 1). 

**Table 1.** Predefined Criteria of Articles

Inclusion Criteria	Exclusion Criteria
**Original research or meta-analysis**	Non-English/non-Chinese publications
**Focus on CD-related intestinal fibrosis**	Case reports or editorials
**Human/animal model studies**	Studies without fibrosis assessment
**TCM/Western medicine interventions**	Duplicated data or incomplete outcomes

Abbreviations: CD, Crohn's disease; TCM, traditional Chinese medicine.

## 2. Pathogenic Mechanisms of Intestinal Fibrosis in Crohn's Disease

### 2.1. Inflammatory Response and Fibrosis

**Figure 1.**
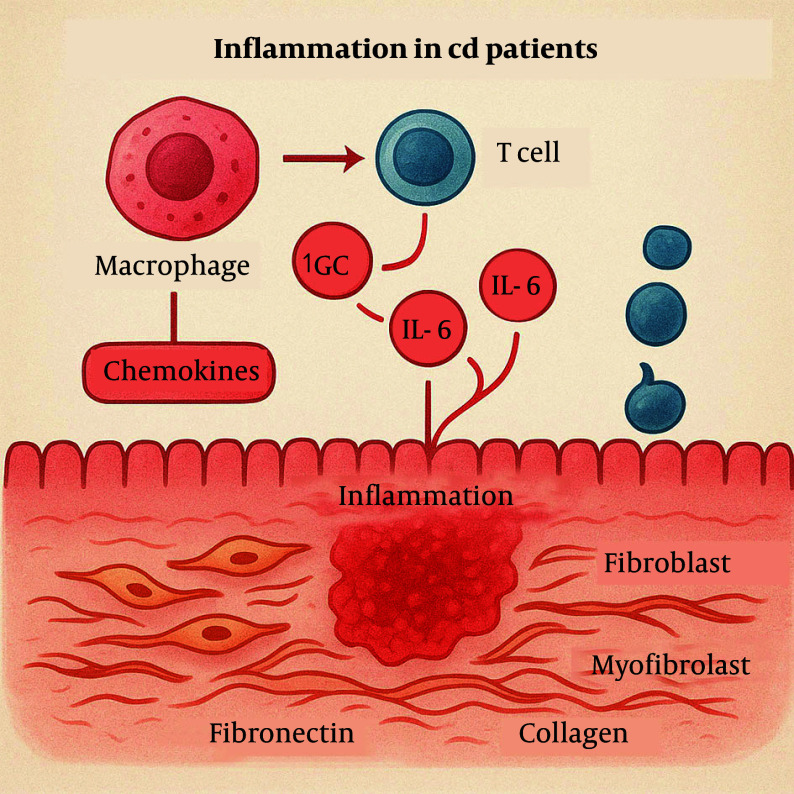
Abnormal immune activation triggers intestinal inflammation through excessive cytokine release and immune cell recruitment, which drives fibroblast activation and excessive extracellular matrix (ECM) deposition, leading to fibrous tissue buildup.

In CD patients, aberrant immune activation drives intestinal inflammation, characterized by excessive cytokine release [e.g., tumor necrosis factor-alpha (TNF-α), interleukin-6 (IL-6)] and recruitment of inflammatory cells. The inflammatory response involves the release of various cytokines, such as TNF-α, interleukin-1 beta (IL-1β), IL-6, and chemokines, which attract and activate inflammatory cells, including macrophages and T-cells (6). Pro-inflammatory factors not only initiate localized inflammation but also stimulate fibroblasts to transform into myofibroblasts. These myofibroblasts secrete large amounts of extracellular matrix (ECM) components, such as collagen and fibronectin, resulting in the accumulation of fibrous tissue (Figure 1) (7).

### 2.2. Activation of Fibroblasts and Extracellular Matrix Deposition

**Figure 2.**
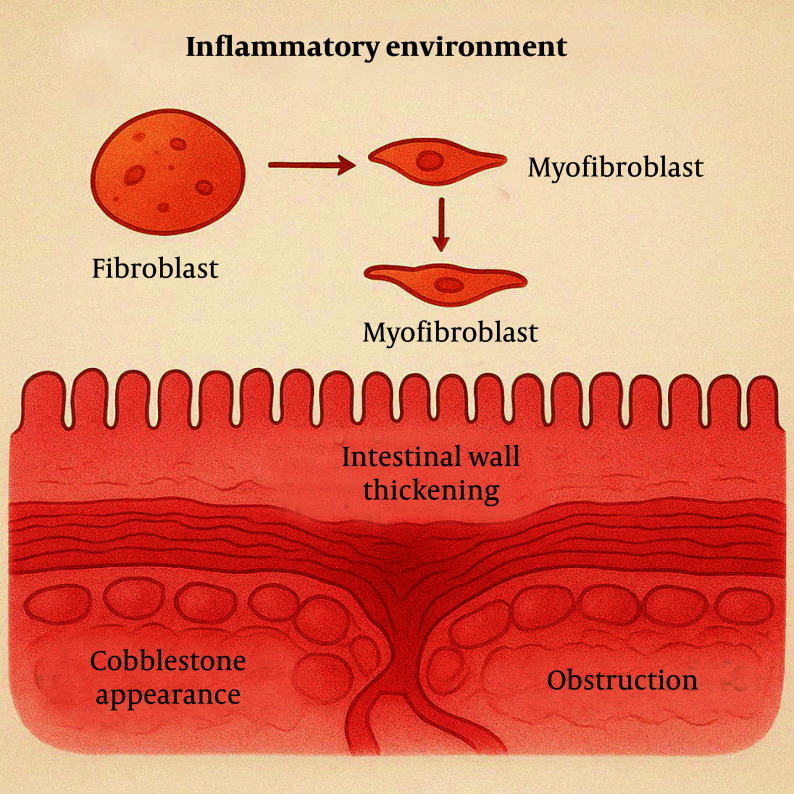
Inflammation activates fibroblasts into myofibroblasts, which produce excess extracellular matrix (ECM). This causes intestinal wall thickening, a cobblestone appearance, and can lead to stricture or obstruction.

In an inflammatory environment, fibroblasts are activated and differentiate into myofibroblasts, a process typically mediated by transforming growth factor-beta (TGF-β) (6). Myofibroblasts possess the ability to synthesize significant amounts of ECM, including collagen types I, III, and V. Aberrant ECM deposition leads to intestinal wall thickening, with hyperplasia of the muscularis mucosa and propria, giving rise to the characteristic "cobblestone" appearance (8). These structural changes ultimately result in intestinal stricture and, in severe cases, obstruction (Figure 2). 

### 2.3. Imbalance Between Apoptosis and Proliferation

**Figure 3.**
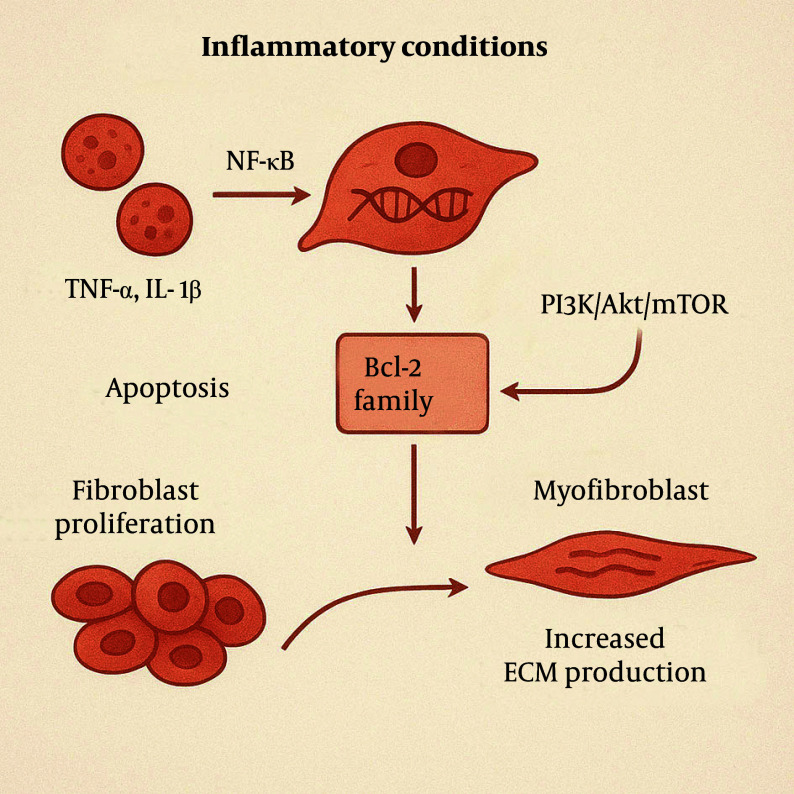
Inflammatory mediators activate survival pathways in fibroblasts, preventing apoptosis and promoting proliferation. These cells differentiate into myofibroblasts, producing excess extracellular matrix (ECM) such as collagen and fibronectin.

Under inflammatory conditions, elevated levels of certain inflammatory mediators, such as TNF-α and IL-1β, activate transcription factors like nuclear factor-kappa B (NF-κB). This activation increases the expression of anti-apoptotic genes, such as members of the Bcl-2 family, enabling fibroblasts and myofibroblasts to resist apoptosis (9). Additionally, inflammatory mediators can activate signaling pathways, such as PI3K/Akt/mTOR, further promoting cell survival and inhibiting apoptosis. These mediators further promote fibroblast proliferation by activating cell cycle progression. These proliferating fibroblasts subsequently differentiate into myofibroblasts, which exhibit enhanced synthetic capacities to produce large quantities of ECM components, such as collagen and fibronectin (Figure 3). 

**Figure 4.**
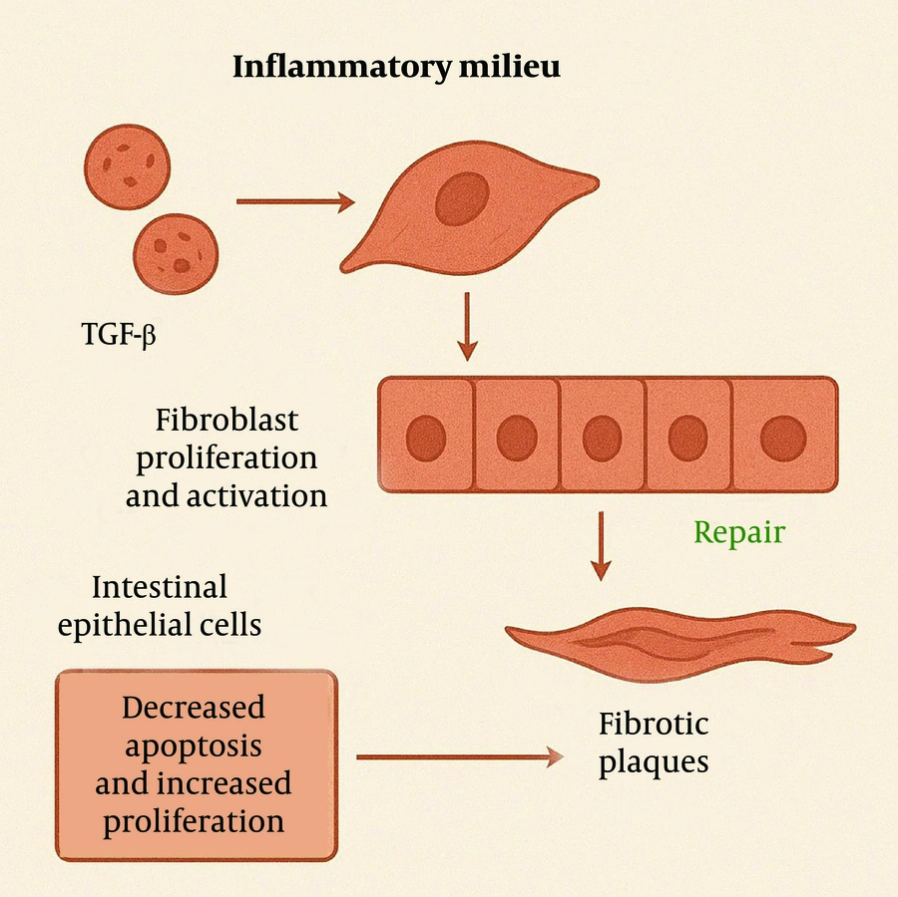
In chronic inflammation, growth factors stimulate fibroblast proliferation and extracellular matrix (ECM) production. Ineffective epithelial repair and reduced apoptosis lead to fibrotic plaque formation.

Growth factors like TGF-β and platelet-derived growth factor (PDGF) are upregulated in the inflammatory milieu, directly promoting fibroblast proliferation and activation (10). To counteract tissue damage, intestinal epithelial cells attempt to repair the affected areas through proliferation and migration. However, in the context of chronic inflammation, this repair process is often ineffective and may even exacerbate local tissue injury. When apoptosis decreases and proliferation increases, ECM produced by fibroblasts accumulates within the tissue, leading to the formation of fibrotic plaques (Figure 4) (11, 12).

Recent studies have highlighted the regulation of apoptosis and proliferation imbalance as a key therapeutic target for fibrosis. For instance, drugs targeting apoptotic pathways, such as Bcl-2 inhibitors, and therapies that suppress fibroblast activation are under active investigation (13).

### 2.4. Non-inflammatory Mechanisms of Fibrosis

Although inflammation serves as a trigger for fibrosis, studies have shown that the progression of fibrosis can occur independently of the inflammatory process. Certain cellular or molecular factors have been implicated in fibrosis, including peroxisome proliferator-activated receptor gamma (PPARγ), toll-like receptor 4 (TLR4), adherent-invasive *Escherichia coli* (AIEC), Th17 immune responses, and plasminogen activator inhibitor-1 (PAI-1) (14-16). Additionally, creeping fat (CrF), a hallmark feature of CD, is considered to play a significant role in fibrosis development (17). Current research suggests that CrF formation is a response to local microbial translocation into the mesentery following intestinal barrier disruption (18-21).

**Figure 5.**
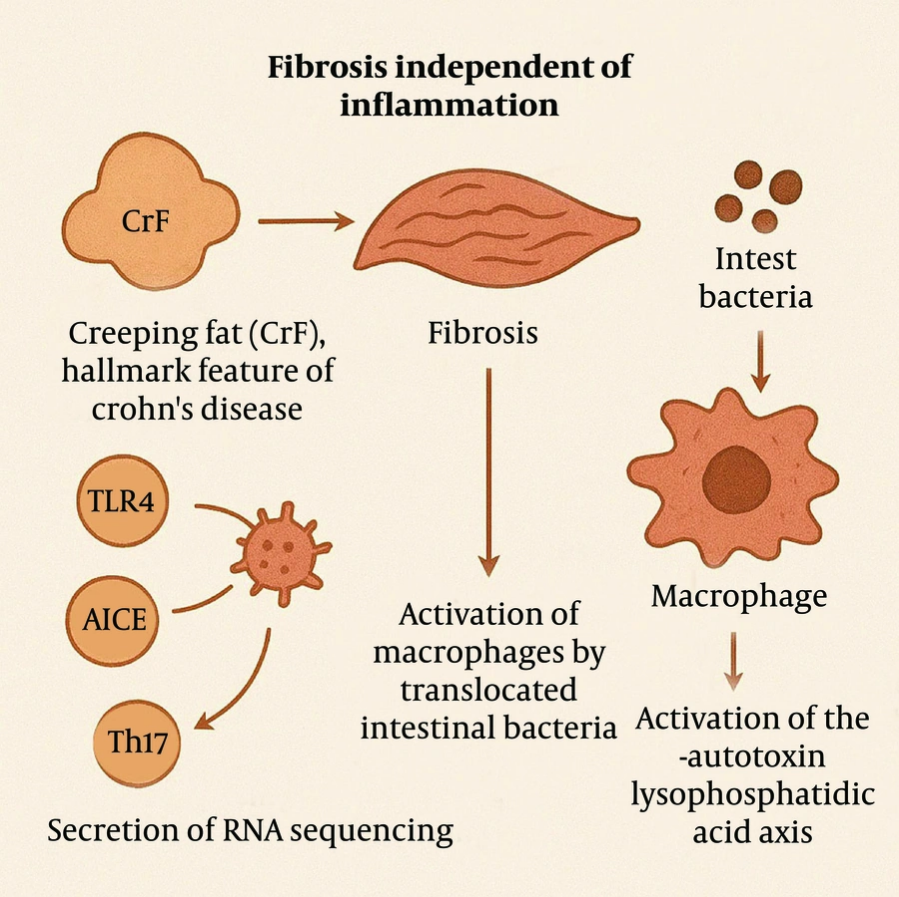
The progression of fibrosis occurs independently of the inflammatory process.

Single-cell RNA sequencing has revealed the presence of both pro-fibrotic and pro-adipogenic signals within CrF. These signals may promote fibrosis through several mechanisms, including activation of macrophages by translocated intestinal bacteria (19), secretion of adipokines that facilitate macrophage transformation (18), activation of the autotoxin-lysophosphatidic acid axis (22), and secretion of free fatty acids (Figure 5) (23-25).

### 2.5. Mucosal Barrier and Microbiota

In CD, the disruption of the intestinal mucosal barrier leads to dysbiosis and the activation of stromal cells. Chronic alterations in the intestinal mucosal barrier and microbiota are critical in the pathogenesis of CD and its associated fibrosis. Damage to the mucosal barrier increases intestinal permeability, allowing bacteria and antigens to traverse the mucosa into the lamina propria. This triggers the activation of immune cells, thereby initiating or exacerbating inflammatory responses and promoting the progression of fibrosis (26).

**Figure 6.**
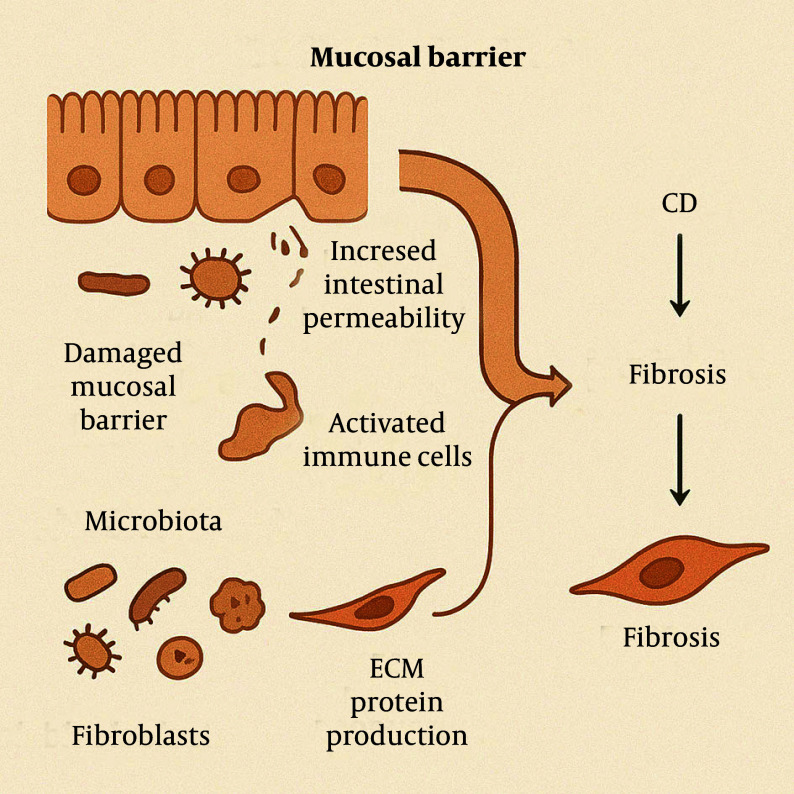
In Crohn’s disease (CD), disruption of the mucosal barrier and gut microbiota imbalance increase intestinal permeability, activate immune and stromal cells, and promote fibroblast-driven extracellular matrix (ECM) production, contributing to fibrosis.

The impairment of the mucosal barrier can manifest as structural and functional abnormalities in tight junction proteins, which disrupt epithelial cell connections and increase intestinal permeability. This heightened permeability may facilitate the progression from IBD to intestinal fibrosis (27). Furthermore, the gut microbiota plays a significant role in CD-related fibrosis. The composition and function of the microbiota can influence the host immune response and inflammatory state, thereby affecting the fibrotic process. For instance, certain bacterial components can activate intestinal fibroblasts and promote the production of ECM proteins, suggesting that specific microbiota-derived molecules may alter the transcriptional state of fibroblasts and contribute to intestinal fibrosis (Figure 6) (28).

### 2.6. Genetic Factors

Genetic factors play a crucial role in the development of intestinal fibrosis, involving not only specific genetic variants but also epigenetic modifications and interactions with the gut microbiota. These factors collectively influence the intestinal inflammatory response and repair processes, ultimately contributing to fibrosis progression. For example, mutations at certain loci in genes such as NOD2, ATG16L1, CX3CR1, IL-23R, and MMP3 have been associated with the fibrotic-obstructive phenotype of CD (29).

**Figure 7.**
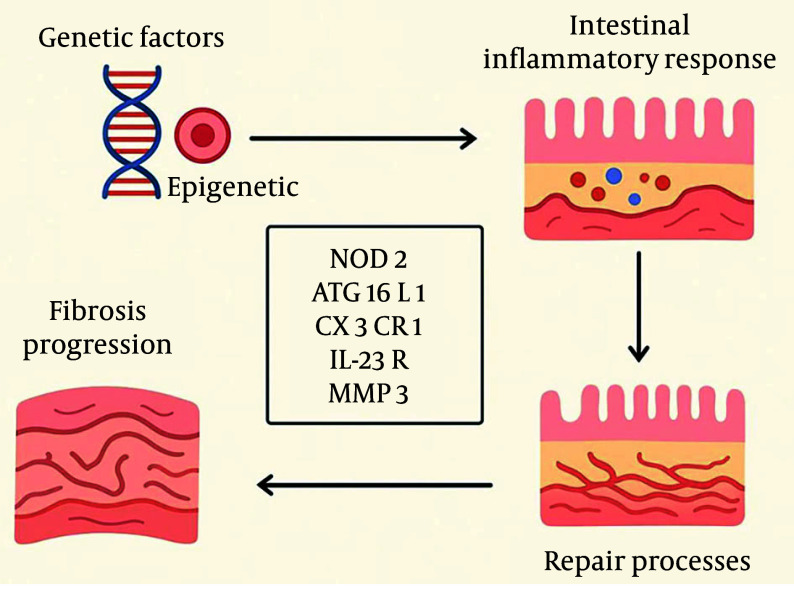
Genetic and epigenetic factors, along with microbiota interactions, influence inflammation and repair in Crohn’s disease (CD). Variants in genes such as NOD2, ATG16L1, and toll-like receptor 4 (TLR4) are linked to fibrosis and strictures.

The NOD2 gene encodes an intracellular pattern recognition receptor that detects bacterial cell wall components, playing a role in the intestinal inflammatory response and fibrosis. Similarly, polymorphisms in the TLR4 gene, which regulates immune responses to bacterial stimuli, are linked to an increased risk of fibrotic strictures in CD (Figure 7) (30).

## 3. Diagnosis of Intestinal Fibrosis

Intestinal fibrosis in CD primarily manifests as intestinal strictures, which may result in varying degrees of obstruction symptoms. Currently, intestinal fibrosis can be assessed using various imaging modalities, including computed tomography enterography (CTE), magnetic resonance enterography (MRE), endoscopy, and ultrasound.

### 3.1. Computed Tomography Enterography

The CTE enables the evaluation of intestinal wall thickness, mucosal enhancement, bowel wall stratification, mesenteric vascularity, and the extent of lesions, thereby facilitating the assessment of intestinal strictures (31). However, mesenteric vascular proliferation is more strongly associated with tissue inflammation than with fibrosis. Since fibrosis and inflammation often coexist, CTE may not reliably differentiate between fibrotic and inflammatory strictures (32).

### 3.2. Magnetic Resonance Enterography

The MRE provides comparable capabilities to CTE for assessing intestinal strictures, but it is currently considered the most accurate modality for evaluating fibrosis (33). This is because gadolinium enhancement in fibrotic tissue exhibits delayed characteristics compared to its enhancement in inflammation-rich vascular tissues (34). Indicators such as T2-weighted imaging (T2WI) signal intensity, apparent diffusion coefficient values, intestinal motility, and time-dependent bowel wall enhancement may aid in distinguishing fibrosis within stenotic bowel segments (35). However, severe fibrotic strictures often coexist with active inflammation, and low signal intensity on T2WI cannot entirely predict the presence or absence of intestinal fibrosis. Advanced MRI techniques, including diffusion-weighted imaging, dynamic contrast enhancement, intravoxel incoherent motion imaging, and magnetization transfer imaging, offer promising non-invasive methods for quantifying the extent of intestinal fibrosis in CD (36, 37).

### 3.3. Ultrasonography

Conventional ultrasonography has limited utility in differentiating between inflammatory and fibrotic strictures. However, specialized imaging techniques can enhance this differentiation. In ultrasound imaging, CD-related fibrosis typically presents as hyperechoic changes in the submucosa, while hypoechoic bowel wall signals are associated with inflammatory edema and congestion. Contrast-enhanced ultrasonography (CEUS) can evaluate the distribution of microvasculature within the bowel wall, aiding in the differentiation of fibrotic strictures (38).

Studies have demonstrated a significant correlation between the degree of histologically confirmed bowel fibrosis and bowel wall stiffness measured by ultrasound elastography (39). This suggests that ultrasound elastography can predict intestinal fibrosis. Real-time shear wave elastography offers the additional capability of estimating tissue stiffness, enabling more precise grading of fibrotic lesions (40). Ultrasonography also has the advantages of being user-friendly, highly reproducible, and suitable for dynamic monitoring of disease progression. However, its diagnostic accuracy is highly dependent on the operator's skill level.

### 3.4. Endoscopy

Endoscopy allows for direct visualization of intestinal strictures but has limited ability to determine the presence of fibrosis. It is also not particularly useful for diagnosing multifocal strictures (41). Typically, fibrotic strictures appear as narrowed segments without concomitant mucosal inflammation or ulceration. However, when both active inflammation and fibrotic strictures coexist, endoscopy can reveal mucosal inflammation or ulceration at the site of stricture (42).

It is important to note that no single diagnostic method is sufficient to comprehensively assess the extent of CD and intestinal fibrosis. Therefore, a combination of diagnostic techniques is commonly employed in clinical practice. For example, combining MRE and endoscopic ultrasound can provide detailed information on both full-thickness and localized bowel wall involvement. Additionally, combining imaging techniques with biomarker testing allows for a more comprehensive evaluation of disease activity and fibrosis at both macroscopic and microscopic levels.

Furthermore, the application of artificial intelligence (AI) in the diagnosis of CD is gaining increasing attention. By analyzing large volumes of imaging and endoscopic data using machine learning algorithms, AI systems can assist clinicians in more accurately identifying lesions, assessing disease severity, and predicting treatment responses. For instance, one study utilized deep learning algorithms to analyze MRE images, achieving better results than human interpretation in distinguishing between inflammatory and fibrotic strictures, with an accuracy rate of 89.7% (43). The comparison of various diagnostic techniques is shown in Table 2. 

**Table 2.** Comparison of Various Diagnostic Techniques

Modalities	Fibrosis Detection	Limitations	Clinical Utility
**CTE**	★★☆☆☆ (Low)	Tissue inflammation interfering	Not reliable for fibrosis
**MRE**	★★★★☆ (High)	Cost; limited availability	Gold standard for fibrosis
**CEUS**	★★★☆☆ (Moderate)	Operator-dependent	Real-time microvascular assessment
**Endoscopy**	★★☆☆☆ (Low)	Limited ability to determine the presence of fibrosis	Reveal mucosal inflammation or ulceration
**AI-assisted**	★★★★☆ (High)	Requires validation cohorts	Automated stricture classification

Abbreviations: CTE, computed tomography enterography; MRE, magnetic resonance enterography; CEUS, contrast-enhanced ultrasonography; AI, artificial intelligence.

## 4. Treatment of Intestinal Fibrosis

### 4.1. Pharmacological Treatment

Currently, there are no approved drugs that can prevent or reverse CD-related intestinal fibrosis. The primary treatment approach remains the active control of intestinal inflammation (44). Developing effective drugs to inhibit intestinal fibrosis is a major challenge in CD research. In other fibrotic conditions such as liver fibrosis and pulmonary fibrosis, several drugs have advanced to phase 2/3 clinical trials, including the Farnesoid X receptor agonist cilofexor, chemokine receptor inhibitor cenicriviroc, and the Janus kinase inhibitor selonsertib (45). However, research focused on inhibiting intestinal fibrosis is mostly still in the preclinical or animal model stage.

In chronic intestinal inflammation models, *Lactobacillus helveticus* strain producing Hsp65 antigen has demonstrated anti-inflammatory and anti-fibrotic effects. Mice treated with Hsp65 exhibited decreased levels of IL-13 and TGF-β, along with increased IL-10 concentrations, confirming the potential effectiveness of this novel anti-fibrotic strategy (28). In mice with fibrosis induced by TNBS or DSS, oral administration of betulinic acid hydroxamate ester was found to prevent colonic inflammation and fibrosis, significantly lowering both fibrosis and inflammation biomarkers. Additionally, the treated mice showed improved epithelial barrier integrity and wound healing, providing a theoretical foundation for further clinical studies (46). Rho-associated protein kinase inhibitor AMA0825 has been shown to reverse intestinal fibrosis in rats and reduce the release of fibrotic factors in CD biopsy specimens (47). A PPARγ ligand with strong affinity, GED-0507-34, was shown to improve intestinal damage in DSS-treated mice and reduce the expression levels of Acta2, COL1a1, and Fn1. It also decreased fibrosis-specific markers, such as α-SMA and α2 type I collagen, displaying varying degrees of anti-fibrotic effects in animal models (48). These research advancements suggest that the development of anti-intestinal fibrosis drugs is progressing actively, with several drugs and therapeutic strategies being explored in hopes of finding effective methods to block or reverse intestinal fibrosis.

Moreover, gene therapy and stem cell therapy, as emerging treatment strategies, show potential applications in CD-related intestinal fibrosis. However, key issues such as ensuring accurate gene editing, stem cell origin, and safety need to be resolved before these therapies can be widely implemented.

### 4.2. Endoscopic and Surgical Treatment

When intestinal fibrosis worsens and leads to obstructive symptoms that are difficult to relieve or reappear shortly after temporary relief, endoscopic or surgical treatments should be considered. Endoscopic treatments primarily include endoscopic balloon dilation (EBD), endoscopic stent placement, and endoscopic stricturotomy (EST). The EBD is suitable for single, non-complicated strictures (not associated with fistulas, abscesses, atypical hyperplasia, or malignancy) that are less than 5 cm in length. The success rate and clinical efficacy exceed 80% (49).

Endoscopic stent placement involves the use of self-expanding metal stents or biodegradable stents. Some small studies have shown that biodegradable stents have a higher technical success rate and long-term symptom relief (50), while self-expanding metal stents have a technical success rate and obstruction relief rate of 93% and 60.9%, respectively (51). However, a follow-up study lasting over two years found that approximately 41.3% of patients who underwent stent placement required repeat endoscopic treatment or surgical intervention (52), and the relief time after stent placement was relatively short. Spontaneous stent migration or dislodgement is common, limiting its clinical use.

The EST is primarily suitable for strictures of the anal and distal colon less than 7 cm in length. Non-uniform, non-concentric strictures are more suitable for EST treatment, with a technical success rate of up to 100%. Only 15.3% of patients required additional stricture-related surgery within one year after EST (53).

For stenosis of CD, the main surgical procedures include bowel resection and stricturoplasty (54, 55). Bowel resection is suitable for patients undergoing their first bowel surgery and those at low risk for short bowel syndrome. Stricturoplasty is used for patients with multiple strictures, those who have undergone extensive bowel resections, or those with short bowel syndrome, particularly for duodenal or terminal ileal strictures, as it maximizes bowel preservation (56). The comparison of treatment strategies for CD-related intestinal fibrosis is shown in Table 3. 

**Table 3.** Comparison of Treatment Strategies for Crohn’s Disease-Related Intestinal Fibrosis

Treatment Strategies	Mechanism/Features	Advantages	Limitations
**Anti-fibrotic drug development (experimental)**	Targeting fibrosis pathways (e.g., FXR agonists, CCR inhibitors, JAK inhibitors); mostly in preclinical/early trials	Inspired by success in liver/lung fibrosis; multiple candidate drugs being tested	No approved drugs for intestinal fibrosis; efficacy/safety in humans unclear
**Probiotics (e.g., ** ***Lactobacillus ** **helveticus*** ** producing Hsp65)**	Anti-inflammatory, IL-13 and TGF-β reduction, increase of IL-10	Non-invasive, microbiota-targeted; showed anti-fibrotic effects in animal models	Limited to animal data; clinical efficacy unproven
**Natural compounds (betulinic acid derivative)**	Colonic inflammation and fibrosis reduction; epithelial barrier repair enhancement	Dual anti-inflammatory and anti-fibrotic effects; animal evidence supportive	No clinical trial data yet
**ROCK inhibitor (AMA0825)**	Intestinal fibrosis reversion; fibrotic factor release reduction	Effective in animal and biopsy studies; potential for reversal, not just prevention	Still experimental; long-term safety unknown
**PPARγ ligand (GED-0507-34)**	Decrease of fibrosis markers (α-SMA, collagen I) and intestinal damage improvement	Demonstrated strong anti-fibrotic effects in preclinical models	No human trials yet
**Gene therapy**	Targeting the modification of fibrosis/inflammation-related genes	Potentially precise and long-lasting	Technical, ethical, and safety challenges
**Stem cell therapy**	Repair promotion and modulating immune response	Potential regenerative effect	Safety, source, and standardization issues unresolved
**EBD**	Mechanical dilation of stricture (< 5 cm, non-complicated)	Success rate > 80%; minimally invasive; repeatable	Risk of recurrence; not suitable for long/multiple strictures
**Endoscopic stent placement**	Self-expanding or biodegradable stents	Provides temporary obstruction relief; biodegradable stents show longer effect	Stent migration common; ~41% need repeat/surgery within 2 y
**EST**	Incision of stricture (< 7 cm, distal colon/anal)	Technical success rate up to 100%; low surgery rate within 1 y (15.3%)	Limited to certain stricture types/locations
**Surgery; bowel resection**	Removal of fibrotic bowel segment	Effective for first surgery, low short bowel risk	Risk of recurrence; may lead to short bowel if repeated
**Surgery; stricturoplasty**	Widening of strictures without resection	Preserves bowel length; useful in multiple strictures or prior resections	Technically demanding; not suitable for all stricture sites

Abbreviations: TGF-β, transforming growth factor-beta; PPARγ, peroxisome proliferator-activated receptor gamma; EBD, endoscopic balloon dilation; EST, endoscopic stricturotomy.

## 5. Traditional Chinese Medicine in the Prevention and Treatment of Intestinal Fibrosis

### 5.1. Traditional Chinese Medicine's Understanding of Intestinal Fibrosis

In traditional Chinese medicine (TCM), there is no specific term for intestinal fibrosis. However, based on the symptoms caused by intestinal fibrosis, such as intestinal narrowing, it can be classified under categories like "accumulation" and manifests clinically as abdominal masses, with pain or distension. The blockage of the intestines and meridians by external pathogens results in the stagnation of Qi, leading to pain when blocked. When Qi stagnation impedes the circulation of blood, it causes blood stasis, depriving the intestines of nourishment, which may lead to ulcers. As blood stagnation worsens over time, accumulation gradually forms.

During the recurrent and chronic phases of the disease, various external pathogens interact with the Qi and blood in the intestines, exacerbating the stasis, making it difficult to resolve. This leads to the stagnation of blood, preventing new blood from being generated. This is the pathological foundation for the development of Qi stagnation and blood stasis in CD. The stagnation of Qi and the accumulation of blood not only exacerbate the condition but also interact with each other, accelerating the accumulation. The transition from deficiency to excess, and from excess to deficiency, runs through the chronic inflammation-to-fibrosis transformation process. Factors such as Qi stagnation, blood stasis, phlegm dampness, and abdominal masses are key pathological contributors to fibrosis. Therefore, eliminating these factors is the primary approach in TCM treatment for fibrosis-related diseases (57-59).

### 5.2. Research Progress in the Prevention and Treatment of Intestinal Fibrosis with Traditional Chinese Medicine

The TCM has shown some success in the prevention and treatment of fibrosis in organs such as the liver, lungs, and kidneys. For instance, compound medicines like Fufang Biejiaruangan Pian, Fuzheng Huayu Pian, and Anluo Huaxian Wan have been demonstrated to exert anti-fibrotic effects through mechanisms such as regulating immune responses, antioxidant properties, and inhibiting the activation of hepatic stellate cells (60).

Some flavonoid-based Chinese herbal monomers, such as hesperidin, hydroxy-safflor yellow A, dendrotoxin, rhodiola rosea glycosides, and ginsenosides, exhibit anti-fibrotic effects in liver and kidney tissues by acting through antioxidant mechanisms and inhibiting TGF-β1 and connective tissue growth factors (61-65).

However, research on the use of TCM for the prevention and treatment of intestinal fibrosis is still in its early stages. Scholars like Zhang Huixiang (66) have observed the effects of total flavonoids from *Hibiscus mutabilis* flowers on the synthesis of type I collagen by intestinal fibroblasts in rats. They found that total flavonoids from *H. mutabilis* could inhibit the generation of type I collagen by activating the AMPK/mTOR pathway to regulate autophagy, thereby improving intestinal fibrosis. However, this study lacked translational validation in human tissues.

Xu Su (67) used a modified Sanleng Wan to treat CD-related intestinal fibrosis and found that this TCM formula could improve clinical outcomes of CD-related intestinal stenosis. But the clinical trials had a small sample size (n = 36) and short follow-up (3 months), limiting statistical power. Further rat experiments demonstrated that Sanleng Wan promoted the production of PPARγ and reduced the levels of intestinal fibrosis-related factors, thereby exerting anti-fibrotic effects (68).

Network pharmacology approaches revealed that Qingchang Tongluo Decoction inhibited the TGF-β1/Smad/VEGF pathway and reduced fibrosis markers in TNBS-induced rats, but this research lacked purified active compounds and long-term safety and human data (69). Xue-Jie-San (XJS) as a single formula showed potential to prevent intestinal fibrosis by blocking Notch 1 and FGL 1 signaling pathways (70). Yet mechanistic studies predominantly rely on DSS/TNBS-induced murine models, which may not fully recapitulate human CD pathophysiology. The comparison of these studies is shown in Table 4. 

**Table 4.** Traditional Chinese Medicine in Anti-fibrosis Reseach

Medicine/Formula	Target/Mechanism	Advantages	Limitations
**Fufang Biejiaruangan Pian, Fuzheng Huayu Pian, Anluo Huaxian Wan**	Regulating immune response, antioxidant, inhibit hepatic stellate cell activation	Proven anti-fibrotic effects in liver/lung/kidney; multi-target	Evidence mainly from non-intestinal fibrosis; limited CD-specific data
**Flavonoids (hesperidin, hydroxy-safflor yellow A, dendrotoxin, rhodiola glycosides, ginsenosides)**	Antioxidant effects, inhibition of TGF-β1 and connective tissue growth factors	Clear molecular targets; testing in liver/kidney	Lack of intestinal fibrosis-specific evidence
**Total flavonoids of ** ***Hibiscus ** **mutabilis***	Activating AMPK/mTOR pathway, regulating autophagy, inhibition collagen I synthesis	Showing inhibition of intestinal fibroblast collagen production	No human tissue validation; only rat fibroblast data
**Modified Sanleng Wan**	Promoting PPARγ, fibrosis-related factors reduction	Clinical trial in CD patients (n = 36) showing benefit; supported by animal studies	Small sample size, short follow-up (3 months); limited statistical power
**Qingchang Tongluo decoction**	Inhibition of TGF-β1/Smad/VEGF pathway, fibrosis markers reduction	Network pharmacology + animal data; multi-target regulation	Active compounds not purified; lack of long-term safety and human studies
**XJS**	Blocking Notch1 and FGL1 signaling	Showing fibrosis prevention in animal models	Evidence only from DSS/TNBS-induced mice; need of human validation

Abbreviations: CD, Crohn's disease; TGF-β, transforming growth factor-beta; PPARγ, peroxisome proliferator-activated receptor gamma; XJS, Xue-Jie-San.

These studies suggest that TCM compound formulas or single components may serve as potential treatments for intestinal fibrosis. However, translating experimental results into clinical applications still requires further exploration. Future TCM research should prioritize randomized controlled trials (RCTs), standardized fibrosis biomarkers, and multi-center validation to mitigate selection bias.

## 6. Summary and Outlook

Intestinal fibrosis in CD remains a significant challenge in the diagnosis and treatment of the condition. In recent years, research on the diagnosis and treatment of CD has gained significant momentum. With the advancement of multi-omics, gut microbiota, cytokines, and other related fields of study, the mechanisms underlying the occurrence and development of intestinal fibrosis have gradually been revealed. However, effective prevention and reversal of intestinal fibrosis remain to be fully explored.

Clinically, a combined approach integrating both TCM and Western medicine may provide an effective solution to the challenge of CD-related intestinal fibrosis. By combining the strengths of both medical systems, such an approach could help control inflammatory activity while preventing or alleviating the progression of fibrosis, thus achieving a comprehensive treatment strategy that addresses both the symptoms and root causes of the disease.

Future research on integrated treatment strategies should focus on deepening our understanding of the disease mechanisms, developing more precise diagnostic and treatment methods, and exploring the molecular and cellular mechanisms of TCM. Additionally, large-scale, long-term clinical studies are needed to further validate the effectiveness and safety of such approaches.

## Data Availability

All data generated or analysed during this study are included in this. Further enquiries can be directed to the corresponding author.
